# Speaking up under constraints: a configurational JD-R explanation of safety voice among young construction workers

**DOI:** 10.3389/fpubh.2026.1886471

**Published:** 2026-07-14

**Authors:** Lili Jia, Wenhao Zhai, Qing Zhu, Sainan Lyu, Peng Cui

**Affiliations:** 1 School of Civil Engineering, Hefei University of Technology, Hefei, Anhui, China; 2 School of Civil Engineering, Nanjing Forestry University, Nanjing, Jiangsu, China; 3 Department of Applied Physics and Electronics, Umea University, Umea, Sweden

**Keywords:** construction safety, fsQCA, job demands-resources theory, psychological safety, safety voice, voice cost, young workers

## Abstract

**Introduction:**

Young construction workers are exposed to high levels of occupational safety risk while often occupying relatively vulnerable positions in work groups. Safety voice is an important participatory mechanism for early hazard identification and occupational injury prevention. However, existing studies have mainly examined the net effects of individual predictors, offering limited insight into how multiple job demands, job resources, and personal capabilities combine to enable or constrain safety voice.

**Methods:**

Drawing on the job demands-resources framework, this study adopted a configurational perspective to examine how different combinations of job insecurity, perceived voice cost, psychological safety, safety empowering supervision, job control, and political skill are associated with high and low safety voice among young construction workers. Survey data from 240 young construction workers in China were analyzed using fuzzy-set qualitative comparative analysis.

**Results:**

The findings reveal multiple configurations leading to high safety voice, indicating that no single condition is sufficient on its own. Instead, safety voice emerges when motivational permission, behavioral discretion, supervisory support, and interpersonal capability are jointly present or compensate for selected demand-side constraints. The configurations associated with low safety voice are not simple opposites of those producing high safety voice, suggesting clear causal asymmetry.

**Discussion:**

This study extends occupational safety and public health research by showing how safety voice among young construction workers depends on configurations of work-related demands, resources, and personal capabilities. The findings provide practical implications for injury prevention, participatory safety management, and workplace health promotion in high-risk construction settings.

## 1 Introduction

Frontline workers are often among the first to encounter or observe emerging hazards during daily site operations. However, their firsthand observations may not be promptly captured by formal safety management systems. This paradox is especially consequential in construction, where dynamic site conditions, physically dangerous tasks, fragmented subcontracting arrangements, and strong reliance on frontline judgment make timely upward safety communication critical for accident prevention (1, 2). Accident statistics across different countries continue to show that construction remains a high-risk industry. Japan reported more than 26,000 construction-site accidents in 2022, Malaysia recorded 8,994 accident cases in 2023, and construction accounted for the highest number of workplace accidents in the European Union during 2008–2020 (3). These persistent risks indicate that construction safety cannot depend only on formal rules, inspections, or post-incident learning. It also depends on whether frontline workers raise concerns before hazards develop into accidents.

Safety voice, defined as workers' upward communication of safety concerns, hazard information, and improvement suggestions, is therefore a critical proactive safety behavior in construction (4, 5). Prior research shows that safety voice enables organizations to identify latent risks and implement corrective actions in a timely manner (6, 7). However, workers may remain quiet even when they recognize unsafe conditions, because speaking up can expose them to interpersonal tension, reputational cost, supervisory disapproval, or employment-related uncertainty (8, 31). Explaining safety voice therefore requires attention not only to whether workers recognize risk, but also to whether they feel permitted, capable, and sufficiently protected to communicate concerns upward.

This issue is particularly salient for young construction workers. Young workers often have shorter tenure, less task-specific experience, weaker job knowledge, and lower authority over work arrangements. Although they may encounter hazards directly in daily site work, these disadvantages may reduce their confidence and perceived legitimacy to communicate safety concerns upward (18, 65). Prior studies suggest that young workers may hesitate to speak up because of newcomer status, limited confidence, fear of being perceived as troublesome, and concern about negative reactions from supervisors or employers (9–11). At the same time, young workers should not be treated as silent by default. Their willingness to voice safety concerns depends on the conditions in which they work, the signals they receive from supervisors, the discretion they have over work-related action, and the interpersonal capability they possess to raise concerns appropriately.

Existing safety voice research has identified important antecedents such as leadership, safety climate, psychological safety, coworker support, perceived risk, job control, and political skill (6, 44, 58). However, most studies remain dominated by variable-centered approaches that estimate the average net effect of individual predictors. Although such studies are valuable, they are less suited to explaining safety voice in construction settings, where workers rarely experience one isolated condition at a time. Young construction workers rarely make safety voice decisions under a single isolated condition. Instead, they simultaneously experience multiple enabling and constraining conditions, including employment insecurity, perceived voice cost, supervisory support, psychological safety, job control, and interpersonal capability (12, 13). Therefore, the net effect of a single variable may not fully explain why some workers speak up whereas others remain quiet. A configurational perspective is more appropriate because it examines how different combinations of demands, resources, and personal capabilities jointly enable or constrain safety voice. Unlike regression, mediation, or moderation models that primarily estimate the net effects of individual predictors, fsQCA captures equifinality, conjunctural causation, and causal asymmetry. It therefore provides additional theoretical value by identifying multiple configurations leading to high and low safety voice.

The Job Demands-Resources (JD-R) framework provides a useful theoretical foundation for organizing these conditions, but it needs to be applied configurationally rather than additively. In the present study, job insecurity and perceived voice cost represent demand-side constraints that increase the perceived risk of speaking up. Psychological safety, safety empowering supervision, and job control represent job resources that provide motivational permission, supervisory legitimacy, and behavioral discretion. Political skill is treated as a personal resource that helps young workers convert safety concerns into socially acceptable upward communication. From this perspective, safety voice is not simply produced by abundant resources or suppressed by high demands. It is more likely to emerge when resources form a mutually reinforcing bundle and when personal capability helps workers act under relational risk.

Accordingly, this study develops a configurational JD-R explanation of safety voice among young construction workers and uses fuzzy-set qualitative comparative analysis (fsQCA) to examine how combinations of job demands, job resources, and personal capability lead to high and low safety voice. Because low safety voice is not equivalent to safety silence as a distinct construct, the analysis of low safety voice is interpreted as identifying configurations associated with constrained or muted upward safety communication rather than directly measuring silence. Three research objectives (*RO*s) guide the study:

*RO*1: To identify key antecedent conditions of safety voice among young construction workers within a configurational JD-R framework.
*RO*2: To examine how different combinations of demands, resources, and personal capability lead to high safety voice.
*RO*3: To examine how different combinations of demands, resources, and personal capability lead to low safety voice, thereby clarifying causal asymmetry between high and low safety voice.

This study makes three contributions. First, it advances safety voice research by reframing safety voice as a configurationally enabled proactive safety behavior rather than the outcome of isolated predictors. Second, it extends JD-R theory by shifting from an additive demand-resource logic to a configurational logic of resource bundling, resource compensation, and bottleneck effects. Third, it identifies political skill as an interpersonal conversion capability that helps young construction workers translate safety concerns into effective upward communication in hierarchical construction settings.

## 2 Literature review

### 2.1 Safety voice in construction

Safety voice refers to change-oriented communication through which employees attempt to improve perceived unsafe working conditions by raising concerns, reporting hazards, or suggesting safer ways of working (4). In construction, safety voice is especially important because the industry remains one of the most hazardous work settings, characterized by dynamic site conditions, physical danger, fragmented teams, and strong dependence on frontline judgment and communication (2, 14). Under such conditions, accident prevention cannot rely on rule compliance alone. It also depends on whether workers identify emerging risks and communicate concerns before incidents occur. Safety voice should therefore be understood as a critical proactive safety behavior rather than a peripheral extra-role action (5, 15).

The importance of safety voice becomes even more important among young workers (9, 16). Young workers are particularly relevant to safety voice research because shorter tenure, limited task-specific experience, and lower workplace authority may increase both their exposure to hazards and their difficulty in raising concerns (17). Evidence that receptive supervisor responses to young workers' safety voice are associated with fewer subsequent injuries further demonstrates its protective value (10). In construction, these challenges are amplified by hazardous tasks, temporary project arrangements, and young workers' limited authority over work conditions (18). Prior studies indicate that they commonly experience bodily injuries, musculoskeletal problems, poorer general health, and heightened mental health risks, partly because many are still undergoing physical, emotional, social, and professional development and often have less authority over their work conditions, strengthening the practical importance of encouraging timely hazard communication and active safety participation among younger workers in construction (19).

Despite its importance, however, studies describe many young workers as taking a “wait-and-see” approach instead of voicing concerns when facing unsafe situations (11). This reluctance is not simply a matter of carelessness but overlapping constraints such as limited hazard awareness, feelings of powerlessness, newcomer status, fear of being seen as troublesome, and concern about making a bad impression on supervisors or employers (6, 20). At the same time, Turner et al. (9) found that adult workers could be less likely than younger workers to speak up when supervisors sent unclear signals about their commitment to safety which suggests that the key issue is not merely whether younger workers speak up less, but under what conditions they are willing, able, and motivated to do so.

For construction research, the key unresolved issue is not simply the recognized importance of safety voice, but the conditions under which young construction workers choose to speak up or remain silent (5). Although prior studies have established that young workers are vulnerable and that safety voice can help prevent harm (9, 20), less is known about how workplace demands, resources, and interpersonal conditions combine to shape this behavior in construction (12). This gap provides a strong basis for adopting a configurational perspective (13).

### 2.2 A configurational JD-R perspective on safety voice

The job demands-resources framework has become one of the most influential models for explaining how work characteristics shape employee strain, motivation, and work-related outcomes. The framework distinguishes between job demands, which require sustained physical, psychological, or social effort and may lead to strain, and job resources, which support goal attainment, reduce demands, and stimulate motivation and development (12, 21–23). Later extensions of the JD-R framework also emphasize the role of personal resources, such as self-efficacy, optimism, and social competence, in shaping how workers interpret and respond to demanding work environments (24, 25). From an occupational health perspective, this framework is particularly useful because excessive job demands and insufficient resources are closely associated with psychosocial hazards, psychological strain, impaired work functioning, and safety outcomes (26–28).

Safety voice can be understood as a behavioral outcome of this demand-resource process. Speaking up about safety concerns requires workers to notice hazards, judge whether communication is worthwhile and take the interpersonal risk of challenging unsafe practices or raising concerns to supervisors. Prior voice research shows that employees often remain silent when they anticipate interpersonal, image-related, or career-related costs, whereas they are more likely to speak up when they perceive psychological safety, supportive leadership, and opportunities for participation (29–32). Safety voice is even more demanding because it often occurs in high-risk, hierarchical, and time-pressured work settings where workers must balance injury prevention against relational and employment-related risks (4, 6, 10). Thus, the JD-R framework provides an integrative logic for organizing both inhibiting and enabling conditions of safety voice: job demands may increase the perceived personal cost of speaking up, whereas job resources may legitimize upward communication and provide workers with the discretion and confidence needed to act.

However, safety voice may not be adequately explained by the independent net effects of single demands or resources. As a socially risky proactive safety behavior, it requires several functional conditions to be aligned. Workers need motivational permission to believe that raising safety concerns is acceptable and will not trigger embarrassment, blame, or interpersonal punishment; behavioral discretion to act on safety judgments rather than remain passive; interpersonal conversion capability to frame concerns appropriately, choose suitable timing, and communicate upward without unnecessarily damaging workplace relationships; and sufficient protection against risk constraints that may make speaking up appear personally costly or unwise. In this sense, safety voice depends not only on whether isolated demands or resources are present, but also on whether multiple constraints, resources, and capabilities form a supportive configuration. This configurational logic extends conventional JD-R thinking in two ways. First, it highlights resource bundling. Consistent with conservation of resources theory, resources often accumulate and operate together as resource caravans rather than functioning in isolation (33–36). For young construction workers, different forms of work-related and personal resources may need to operate jointly to transform risk awareness into actual safety voice. Second, it highlights compensation and bottleneck effects. High job demands do not necessarily eliminate safety voice if strong resources and personal capabilities compensate for selected constraints, whereas weak resources may suppress voice even when perceived demands are not highly salient. Such conjunctural, compensatory, and asymmetric patterns are difficult to capture through variable-centered models, which mainly estimate average net effects, but are well suited to configurational approaches such as fsQCA. FsQCA allows researchers to identify conjunctural causation, equifinality, and causal asymmetry, making it appropriate for examining multiple pathways to high and low safety voice (13, 37–40).

### 2.3 Antecedent conditions and configurational propositions

Building on the functional logic developed above, this study identifies six antecedent conditions that operationalize the configurational JD-R perspective in the context of young construction workers' safety voice. Job insecurity and perceived voice cost are selected to represent risk constraints; psychological safety and safety empowering supervision represent motivational permission; job control represents behavioral discretion; and political skill represents interpersonal conversion capability. These conditions jointly capture the demand-side constraints, job resources, and personal capability that may combine to enable or suppress safety voice.

Job insecurity and perceived voice cost are treated as demand-side constraints. Job insecurity reflects uncertainty about continued employment and may discourage workers from raising concerns when voice is perceived as threatening future opportunities (16, 41). This condition may be especially salient for young construction workers because they often occupy more temporary, peripheral, or probationary positions and may have limited bargaining power in project-based employment settings. Perceived voice cost captures anticipated interpersonal, image-related, or relational risks associated with speaking up, such as being viewed as troublesome, damaging relationships with supervisors or coworkers, or creating a negative impression (29, 42). Both conditions increase the perceived downside of safety voice and may make silence appear safer than speaking up.

Psychological safety, safety empowering supervision, and job control are treated as job resources. Psychological safety reduces fear of embarrassment, blame, or punishment and helps workers view upward safety communication as acceptable (5, 43). Safety empowering supervision legitimizes participation by encouraging suggestions, inviting involvement, and responding constructively to safety concerns (7, 8). This resource is particularly important in hierarchical construction settings because supervisors often control access to information, task arrangements, and informal judgments about whether workers' concerns are welcomed. Job control provides discretion and agency, allowing workers to act on safety judgments rather than merely comply with instructions (44). Together, these resources create a supportive environment in which young workers are more likely to regard safety voice as both acceptable and feasible.

Political skill is treated as a personal resource. In this study, political skill refers to professional interpersonal communication capability rather than ingratiation or manipulative impression management. It reflects the ability to understand social situations, manage impressions, and communicate effectively under relational risk (51, 58). In this study, political skill is not treated as manipulative social competence, but as an interpersonal communication capability that helps young workers translate safety concerns into acceptable and actionable upward communication. In hierarchical construction settings, especially in China where authority sensitivity and relational norms may shape upward communication, political skill can help young workers decide when, how, and to whom safety concerns should be expressed (45). It therefore functions as a conversion resource that turns recognized safety concerns into feasible safety voice behavior.

The six conditions were not intended to exhaust all possible determinants of safety voice, but were selected to capture the core functional components of a configurational JD-R explanation: demand-side constraints, job resources, and personal capability. Other contextual and relational factors, such as team cohesion, coworker support, safety climate, and external labor market scarcity, may also shape safety voice and could be examined in future configurational research. Within the scope of the present study, these conditions are expected to operate interdependently rather than as isolated predictors; therefore, no single condition is assumed to independently determine safety voice. Instead, high and low safety voice are expected to emerge from different configurations of demand-side constraints, job resources, and personal capability. Because fsQCA does not test linear, one-directional causal relationships in the same way as regression-based methods, this study uses propositions rather than conventional hypotheses. These propositions specify configurational expectations concerning equifinality, resource bundling and compensation, constrained low safety voice, and causal asymmetry. They are intended to guide the interpretation of sufficient configurations rather than to predict the independent net effect of any single antecedent condition. The propositions are formulated as follows:

P1 (Equifinality). High safety voice among young construction workers can be produced by multiple distinct configurations of job demands, job resources, and personal capability.
P2 (Resource bundling and compensation). Strong job and personal resources may jointly enable high safety voice and may compensate for selected demand-side constraints.
P3 (Constrained low safety voice). Low safety voice is likely to arise from specific combinations of weak resources, limited interpersonal capability, and demand-side constraints, rather than from any single condition alone.
P4 (Causal asymmetry). The configurations associated with high safety voice are not expected to be the mirror image of those associated with low safety voice.

## 3 Research methods

### 3.1 Research design

#### 3.1.1 Research design and sample

This study adopts a configurational research design grounded in the JD-R framework and based on questionnaire data collected from young construction workers in China. This design is appropriate because safety voice is unlikely to be shaped by isolated antecedents alone; rather, it is more likely to emerge from combinations of job demands, job resources, and personal or contextual conditions. Accordingly, this study focuses on identifying how multiple antecedent conditions jointly contribute to high and low levels of safety voice among young construction workers.

The target population comprised young construction workers aged 35 years or below, including frontline skilled workers, technical personnel, and junior-level site supervisors. Data were collected through a nationwide online questionnaire survey conducted between March and May 2024. Respondents were contacted with the assistance of project managers, site engineers, and industry contacts, who distributed the online questionnaire to eligible young construction workers. Participation was voluntary and anonymous. Before completing the questionnaire, respondents were informed of the academic purpose of the study, the confidentiality of their responses, and their right to withdraw at any time. No personally identifiable information was collected. A total of 357 questionnaires were distributed across multiple construction projects in China, covering different project types such as building construction, infrastructure construction, and municipal engineering. After excluding incomplete and invalid responses, 240 valid questionnaires were retained for analysis, resulting in an effective response rate of 67.2%. Although a larger sample would be desirable for estimating national trends, the purpose of this study was to identify configurational pathways rather than to provide a nationally representative prevalence estimate. QCA was originally developed for small-N research but has been extended to large-*N* settings as an alternative or complement to regression analysis (46). Recent methodological guidelines also emphasize that QCA is suitable for explaining how configurations of conditions lead to outcomes (47). Therefore, for a six-condition fsQCA design, 240 valid cases provide sufficient empirical diversity for truth-table construction and configurational comparison.

The demographic characteristics of the valid respondents are summarized in Table 1. Educational background and work experience were used as demographic descriptors rather than core configurational conditions. This decision was made because the present study focuses on proximal psychosocial and work-design conditions that directly shape safety voice, including job insecurity, perceived voice cost, psychological safety, safety empowering supervision, job control, and political skill. Nevertheless, education and work experience may influence hazard recognition, self-confidence, and perceived legitimacy to speak up. Future research could examine whether these demographic characteristics operate as boundary conditions or additional configurational conditions in shaping safety voice among young construction workers.

**Table 1:** Demographic characteristics of valid respondents (*n* = 240).

Variable	Category	Frequency	Percentage (%)
Gender	Male	187	77.9
	Female	53	22.1
Age	Below 20 years old	22	9.2
	20–24 years old	75	31.3
	25–29 years old	64	26.7
	30–35 years old	79	32.9
Work experience	< 1 year	48	19.9
	1–5 years	91	38.1
	6–10 years	46	19.3
	11–15 years	25	10.4
	>15 years	30	12.3
Educational background	Primary school or below	9	3.6
	Junior high school	26	10.9
	Senior high school	30	12.3
	Junior college	43	17.9
	Bachelor's degree	91	38.1
	Postgraduate degree	41	17.1
Project type	Infrastructure projects	85	35.6
	Building construction projects	80	33.3
	Industrial construction	36	14.8
	Agricultural and water conservancy projects	7	3.1
	Other projects	32	13.2

To reduce project-specific bias, respondents were recruited from different companies, project sites, project types, and locations rather than from a single organization or project. This strategy increased contextual diversity and reduced the likelihood that the identified configurations reflected the safety culture of one specific project.

#### 3.1.2 Variable selection

The selection of antecedent conditions was guided by the JD-R framework. Job insecurity and perceived voice cost were classified as job demands because they capture employment-related uncertainty and anticipated interpersonal or image-related risks associated with speaking up. Job control, safety empowering supervision, and psychological safety were classified as job resources because they reflect discretion at work, supervisory encouragement, and a safe interpersonal environment for expressing concerns. Political skill is conceptualized as a personal resource with an interpersonal conversion function. Within the JD-R framework, it qualifies as a personal resource because it represents a relatively stable individual capability that helps workers understand social cues, manage interpersonal risk, and communicate effectively. In the context of safety voice, this resource operates as a conversion capability because it enables young workers to translate safety concerns into acceptable and actionable upward communication. Table 2 summarizes the selected antecedent conditions and their theoretical roles.

**Table 2:** Literature-based selection and JD-R classification of antecedent conditions.

Variable	Definition	JD-R role	Representative literature	Relevance to the present study
Job insecurity	The perceived uncertainty or threat concerning the continuity and stability of one's current employment.	Job demand	Paterson et al. (16); Salman et al. (41)	Employment uncertainty; fear of negative consequences; reluctance to speak up
Perceived voice cost	The anticipated interpersonal, reputational, or career-related consequences associated with expressing concerns or suggestions.	Job demand	Detert and Edmondson (29); Tucker and Turner (10)	Anticipated relational risk; image cost; potential career penalty
Job control	The degree of discretion and influence workers have over their tasks, work methods, and work-related decisions.	Job resource	Curcuruto et al. (44); Mathisen et al. (63)	Work discretion; decision latitude; capacity to act on safety concerns
Safety empowering supervision	Supervisory behavior that encourages workers' participation, involvement, and autonomy in identifying and addressing safety issues.	Job resource	Jada and Mukhopadhyay (64); Curcuruto and Griffin (8); Yu et al. (7)	Supervisory encouragement; voice legitimation; supportive leader response
Psychological safety	The belief that expressing concerns, questions, or different opinions is safe and will not result in interpersonal punishment or embarrassment.	Job resource	Edmondson (43); Sun et al. (5)	Safe interpersonal climate; low fear of speaking up; tolerance for expressing concerns
Political skill	The ability to understand social situations and influence others through effective, appropriate, and seemingly sincere interpersonal behavior.	Personal resource	Sun et al. (58); Dai et al. (45)	Interpersonal influence; social astuteness; upward communication capability

#### 3.1.3 Measures

All constructs were measured using previously validated scales (Table 3). Unless otherwise stated, responses were recorded on a five-point Likert scale ranging from 1 (“strongly disagree”) to 5 (“strongly agree”). Job insecurity was measured using four items from de Witte (48). Perceived voice cost was measured using four items adapted from Detert and Edmondson (29). Psychological safety was measured using five items from Liang et al. (31). Safety empowering supervision was measured using four items adapted from Ahearne et al. (49). Job control was measured using three items based on Prümper et al. (50). Political skill was measured using six items from Ferris et al. (51). The items emphasized social astuteness, effective communication, relationship building, and interpersonal sincerity. To reduce social desirability bias, the questionnaire was administered anonymously, confidentiality was assured, and respondents were informed that there were no right or wrong answers. Safety voice was measured using five items from Tucker et al. (4). Before the formal survey was administered, the questionnaire was reviewed for clarity and contextual appropriateness. Minor wording adjustments were made to ensure that the items were understandable to respondents in construction settings while preserving the conceptual meanings of the original scales.

**Table 3:** Measurement items, factor loadings, and reliability statistics.

Measurement item	Factor loading
**Job insecurity** (KMO = 0.784; AVE = 0.664; Cronbach's α = 0.851)	
I feel that I am likely to lose my current job soon.	0.833
I am confident that I will be able to keep my current job. **(R)**	0.807
I feel uneasy about the future of my current job.	0.762
I think I may lose this job in the near future.	0.855
**Political skill** (KMO = 0.865; AVE = 0.527; Cronbach's α= 0.838)	
I am very good at understanding others' motives.	0.852
I am good at building relationships with others easily.	0.682
I am able to communicate with others easily and effectively.	0.722
I have built a network of relationships through which I can get help from coworkers when needed.	0.662
I spend time and effort developing connections and relationships with others.	0.777
When communicating with others, I come across as sincere and genuine.	0.640
**Psychological safety** (KMO = 0.761; AVE = 0.536; Cronbach's α= 0.744)	
In my work team, I can express my true feelings about my work.	0.751
In my team, I can freely express my ideas.	0.715
In my team, expressing one's true thoughts is welcomed.	0.761
Even if I have a different opinion, other members of the team will not deliberately make things difficult for me.	0.556
I worry that expressing my true thoughts at work may have negative consequences for me. **(R)**	0.845
**Safety empowering supervision** (KMO = 0.792; AVE = 0.578; Cronbach's α = 0.797)	
My supervisor discusses with us how to improve safety at work.	0.761
My supervisor spends time helping us learn how to identify potential problems in advance.	0.786
My supervisor encourages us to work safely by explaining the reasons, rather than simply demanding obedience.	0.662
My supervisor often discusses with us the risks that may arise in our work.	0.822
**Job control** (KMO = 0.765; AVE = 0.527; Cronbach's α = 0.785)	
Considering your work as a whole, to what extent can you decide the order of your work steps yourself?	0.733
How much influence do you have over which tasks are assigned to you?	0.705
Can you plan and organize your work independently?	0.739
**Perceived voice cost** (KMO = 0.804; AVE = 0.639; Cronbach's α = 0.838)	
Speaking up about safety issues could negatively affect my relationships at work.	0.774
Speaking up about safety issues could make my supervisor think less favorably of me.	0.767
Speaking up about safety issues could hurt my future opportunities at work.	0.785
Speaking up about safety issues could lead to negative consequences for me.	0.867
**Safety voice** (KMO = 0.843; AVE = 0.510; Cronbach's α = 0.845)	
I make suggestions about how safety can be improved.	0.759
I tell my colleague who is doing something unsafe to stop.	0.708
I discuss new ways to improve safe working with my colleagues or boss	0.740
I inform my supervisor when I notice a potential safety hazard on site.	0.713
I report to my boss if my colleagues break any safety rules.	0.645

#### 3.1.4 Measurement validation

Before conducting fsQCA, the reliability and validity of the measurement scales were examined. Internal consistency was assessed using Cronbach's alpha. As shown in Table 3, Cronbach's alpha values ranged from 0.744 to 0.851, all exceeding the commonly accepted threshold of 0.70, indicating acceptable internal consistency (52). Convergent validity was assessed using average variance extracted (AVE) and factor loadings. The AVE values ranged from 0.510 to 0.664, all above the recommended threshold of 0.50, suggesting acceptable convergent validity (53). The KMO values ranged from 0.761 to 0.865, indicating that the data were suitable for factor analysis (54). The factor loadings ranged from 0.556 to 0.867. Although several factor loadings were below 0.70, the corresponding constructs were retained because their AVE and Cronbach's alpha values met acceptable standards, and the items were theoretically consistent with the original validated scales. Discriminant validity was further examined using the Fornell–Larcker criterion. The square root of AVE for each construct was greater than its correlations with other constructs, indicating acceptable discriminant validity (53). Overall, the measurement results supported the reliability and convergent validity of the constructs used in the subsequent fsQCA analysis.

### 3.2 Data analysis

#### 3.2.1 Rationale for using fsQCA

To examine how combinations of antecedent conditions jointly shape safety voice, this study employed fsQCA as the main analytical approach (37). Compared with conventional variable-centered methods, fsQCA is particularly suitable for examining causal complexity because it accommodates equifinality, conjunctural causation, and causal asymmetry. In other words, it allows multiple distinct combinations of conditions to lead to the same outcome and recognizes that the configurations associated with the presence of an outcome may differ from those associated with its absence (38, 55). This analytical strategy is appropriate for the present study because safety voice among young construction workers is unlikely to be determined by any single antecedent in isolation. Instead, it is more likely to emerge from different combinations of demands, resources, and personal or contextual conditions. In addition to identifying sufficient configurations associated with high and low safety voice, the analysis also examined whether any single condition constituted a necessary condition for the outcome (37).

#### 3.2.2 Calibration and analytical procedures

The fsQCA analysis was conducted in four steps: calibration, necessity analysis, truth table construction, and configurational solution analysis. First, because fsQCA is based on set membership rather than raw variable scores, all antecedent conditions and the outcome variable were calibrated into fuzzy-set membership scores ranging from 0 (full non-membership) to 1 (full membership) (37). Because no widely accepted external substantive thresholds are available for defining full membership, crossover, and full non-membership in the sets examined in this study, percentile-based calibration was adopted. In the absence of external benchmarks, the 75th, 50th, and 25th percentiles provide transparent and replicable anchors that reflect relative differences within the sample. This approach is commonly used in survey-based fsQCA research when constructs are measured using Likert-type scales and substantive thresholds have not been established. Accordingly, the 75th percentile was specified as the threshold for full membership, the 50th percentile as the crossover point, and the 25th percentile as the threshold for full non-membership. The detailed calibration values are reported in Table 4. To avoid ambiguity at the crossover point, cases with fuzzy-set membership scores exactly equal to 0.500 were slightly adjusted by 0.001.

**Table 4:** Summary of variables and calibration values.

Variable	Mean	Std. Dev.	Calibration values at		
			75%	50%	25%
Job insecurity	2.983	1.026	3.75	3.00	2.25
Political skill	3.483	0.753	4.00	3.50	3.00
Psychological safety	3.477	0.728	4.00	3.40	3.00
Safety empowering supervision	3.581	0.865	4.25	3.63	3.00
Job control	3.466	0.788	4.00	3.5	3.00
Perceived voice cost	2.952	0.951	3.50	3.00	2.25
Safety voice	3.618	0.874	4.40	3.60	3.00

A necessity analysis was conducted to determine whether any single antecedent condition constituted a necessary prerequisite for high or low safety voice. In line with established fsQCA practice, a condition was considered necessary when its consistency score exceeded 0.90. Conditions that did not reach this threshold were interpreted as non-necessary, indicating that the outcome could not be explained by any single factor alone.

In addition, a truth table was constructed to list all logically possible combinations of the antecedent conditions. Based on the calibrated membership scores, each case was assigned to a corresponding configuration. To retain only empirically meaningful configurations, a frequency threshold of 3 was applied, consistent with prior fsQCA research using comparable sample sizes. In addition, a raw consistency threshold of 0.85 was used to identify configurations that were sufficiently associated with the outcome (37, 56). PRI consistency was also taken into account to reduce simultaneous subset relations to both the outcome and its negation, and a PRI consistency threshold of 0.70 was adopted.

Configurational solutions for both high safety voice and low safety voice were derived through Boolean minimization. Solution quality was evaluated using raw coverage, unique coverage, solution coverage, and solution consistency. Separate analyses were conducted for high and low safety voice in order to account for causal asymmetry. To assess robustness, the case frequency threshold was further increased from 3 to 4. The main configurational patterns remained substantively stable, which supports the robustness of the findings (57). All analyses were performed using fsQCA 3.0 software.

## 4 Results

### 4.1 Necessity analysis

Before examining configurational sufficiency, necessity analysis was conducted for each antecedent condition and its negation with respect to both high and low safety voice. As shown in Table 5, no single condition reached the 0.90 consistency threshold for either outcome. For high safety voice, the highest consistency values were observed for safety empowering supervision (0.777), psychological safety (0.769), political skill (0.758), and job control (0.755), but all remained below the benchmark. For low safety voice, the highest consistency values were found for the absence of political skill (0.777), the absence of safety empowering supervision (0.774), the absence of job control (0.769), and the absence of psychological safety (0.727), again below the necessary-condition threshold.

**Table 5:** Necessity analysis of single conditions for high and low safety voice.

Conditions	High safety voice		Low safety voice	
	Consistency	Coverage	Consistency	Coverage
Job insecurity	0.549	0.535	0.568	0.573
~Job insecurity	0.561	0.557	0.538	0.552
Political skill	0.758	0.766	0.330	0.345
~Political skill	0.352	0.337	0.777	0.768
Psychological safety	0.769	0.731	0.376	0.370
~Psychological safety	0.352	0.337	0.727	0.765
Safety empowering supervision	0.777	0.769	0.339	0.347
~Safety empowering supervision	0.341	0.333	0.774	0.782
Job control	0.755	0.760	0.344	0.358
~Job control	0.363	0.349	0.769	0.764
Perceived voice cost	0.540	0.506	0.588	0.569
~Perceived voice cost	0.540	0.559	0.490	0.524

These findings indicate that neither high nor low safety voice can be explained by any single antecedent condition. This result supports the configurational assumption that safety voice among young construction workers is shaped by combinations of demand-side constraints, job resources, and personal capability rather than by isolated predictors.

### 4.2 Configurations for high safety voice

The sufficiency analysis for high safety voice identified two configurations, as shown in Table 6. The overall solution coverage was 0.522, and the overall solution consistency was 0.939, indicating that the solution explains a meaningful proportion of cases with high safety voice and that the identified configurations show strong subset relations with the outcome.

**Table 6:** Configurational solutions for high and low safety voice.

Solutions	Model 1		Model 2					
	High safety voice		Low safety voice					
	1	2	1	2	3	4	5	6
Job insecurity		•		⊗	•	•	•	•
Political skill	•	•		⊗	⊗	•	⊗	⊗
Psychological safety	•	•	⊗		⊗			⊗
Safety empowering supervision	•		⊗	⊗		⊗	⊗	
Job control	•	•	⊗	⊗	⊗	⊗	•	•
Perceived voice cost		•	•	⊗	⊗	•	•	•
Raw coverage	0.494368	0.262392	0.399452	0.211817	0.197128	0.126413	0.151471	0.143922
Unique coverage	0.259620	0.027643	0.163832	0.060559	0.046747	0.004779	0.008894	0.009615
Consistency	0.948615	0.930575	0.896765	0.935218	0.904166	0.906377	0.904858	0.870027
Solution coverage	0.522012		0.580293					
Solution consistency	0.939453		0.864702					

•, Core presence; •, Peripheral presence; ⊗, Core absence; ⊗, Peripheral absence; Blank spaces indicate “don't care”.

The first configuration represents a resource-bundling pathway. It combines the core presence of political skill, psychological safety, and job control with the peripheral presence of safety empowering supervision. This configuration has the strongest empirical relevance, with a raw coverage of 0.494, a unique coverage of 0.260, and a consistency of 0.949. The result suggests that high safety voice is most likely when young workers simultaneously possess interpersonal capability, experience psychological safety, and have sufficient discretion over work-related action, while supervisory empowerment further reinforces the pathway.

The second configuration provides a supplementary resource-compensatory pathway under perceived constraints. It combines the presence of job insecurity, political skill, psychological safety, job control, and perceived voice cost. This pathway has a raw coverage of 0.262, a unique coverage of 0.028, and a consistency of 0.931. Because its unique coverage is relatively low, this configuration should be interpreted as an auxiliary rather than dominant pathway. Nevertheless, it suggests that high safety voice may still occur under employment uncertainty and perceived voice cost when strong personal and contextual resources coexist.

Across both configurations, political skill, psychological safety, and job control appear repeatedly. This pattern indicates that high safety voice depends not simply on the availability of one supportive condition, but on a core bundle of interpersonal capability, psychological permission, and behavioral discretion. The two configurations also demonstrate equifinality: high safety voice can emerge through more than one combination of demands and resources.

### 4.3 Configurations for low safety voice

The analysis of low safety voice produced six sufficient configurations. The overall solution coverage was 0.580, and the overall solution consistency was 0.865, indicating that the solution captures a substantial proportion of low-safety-voice cases with acceptable consistency. Because several configurations exhibit relatively low unique coverage, the analysis emphasizes recurring configurational patterns rather than interpreting each minor solution as an equally important pathway.

The first pattern, consisting of Model 2-1, can be described as a high-cost constrained-voice pathway. The most empirically relevant configuration combines the absence of psychological safety, the absence of safety empowering supervision, and the absence of job control with the presence of perceived voice cost. This pathway has the highest raw coverage among the low-safety-voice configurations (0.399), with a unique coverage of 0.164 and a consistency of 0.897. It indicates that young workers are unlikely to voice safety concerns when speaking up is perceived as costly and when they simultaneously lack interpersonal safety, supervisory support, and work discretion.

The second pattern, consisting of Model 2-2 and Model 2-3, reflects a capability- and resource-deficient low-voice pathway. One configuration combines the absence of job insecurity, political skill, safety empowering supervision, job control, and perceived voice cost. Another combines the presence of job insecurity with the absence of political skill, psychological safety, job control, and perceived voice cost. These pathways suggest that low safety voice may arise even when perceived voice cost is not salient, provided that workers lack the interpersonal capability and supportive resources needed to raise concerns effectively.

The third pattern, Model 2-4, Model 2-5, and Model 2-6, reflects a demand-loaded muted-voice pathway under constrained resources. Several configurations combine job insecurity and perceived voice cost with weak enabling conditions such as the absence of safety empowering supervision, psychological safety, job control, or political skill. These pathways indicate that employment uncertainty and anticipated interpersonal costs can intensify low safety voice when workers are already located in a weak-resource environment.

Overall, the low-safety-voice results support causal asymmetry. Low safety voice is not simply the mirror image of high safety voice. Rather, it is generated through distinct constrained-voice configurations involving weak resources, limited interpersonal capability, and, in some cases, stronger demand-side constraints. For robustness assessment, the case frequency threshold was increased from 3 to 4, and the main configurational patterns remained substantively stable. A robustness check using a higher case-frequency threshold produced substantively similar patterns, reducing the likelihood that the main findings reflect statistical noise.

## 5 Discussion

### 5.1 Key findings

#### 5.1.1 Resource configurations for high safety voice

A first key finding is that high safety voice among young construction workers is generated through resource-based configurations rather than through any single antecedent alone. The necessity analysis showed that no individual condition qualified as necessary for high safety voice, while the sufficiency analysis identified two distinct pathways leading to the outcome. Across these pathways, political skill, psychological safety, and job control appeared repeatedly, whereas safety empowering supervision strengthened one of the configurations. This pattern supports the proposition that safety voice is best understood as a configurational outcome shaped by conjunctural causation rather than a simple net effect of isolated variables.

This finding is broadly consistent with prior studies showing that psychological safety reduces the interpersonal risk of speaking up, job control enhances workers' sense of agency, and supportive supervision encourages safety voice (5, 43, 44). This study extends this literature by showing that these factors are most influential when they operate together. In other words, high safety voice is not simply the result of one supportive condition in isolation, but the outcome of a bundled resource structure in which interpersonal safety, behavioral discretion, and communication capability reinforce one another. A possible explanation is that safety voice is both proactive and risky (7). For young construction workers, speaking up requires more than recognizing a hazard. It also requires confidence that voice will be accepted, sufficient room to act on one's judgment, and the ability to present concerns in a socially appropriate way (5, 7). From a configurational JD-R perspective, this suggests that high safety voice is more likely to emerge when multiple enabling resources are assembled simultaneously rather than when only one favorable factor is present (12, 13).

Taken together, these findings support P1, which proposes that high safety voice can be produced by multiple distinct configurations. They also provide evidence for P2, because high safety voice depends on a bundled structure of job and personal resources rather than on any single enabling condition.

#### 5.1.2 Political skill as an enabling condition

Political skill plays an important role in shaping safety voice among young construction workers. In the present study, political skill appeared in both high-safety-voice configurations and was absent in several low-safety-voice pathways. This pattern suggests that political skill is not merely a peripheral interpersonal trait, but a recurrent enabling condition that helps young workers translate safety concerns into actual voice behaviors (58).

This finding is consistent with prior research suggesting that political skill can enhance voice efficacy and help employees choose appropriate timing, targets, and communication strategies when expressing concerns (58, 59). However, compared with much of the existing safety voice literature, our results place greater emphasis on this individual capability. Previous studies have more often highlighted contextual factors such as safety climate, supervisory support, and leadership commitment (6, 7, 60). The present findings do not contradict those studies, but they suggest that for young workers, interpersonal capability may be especially consequential. The importance of political skill is particularly salient for young construction workers because they often occupy relatively low-status positions in hierarchical and relationship-sensitive construction settings. Speaking up about safety may involve challenging supervisors, questioning established practices, or drawing attention to problems that others prefer to ignore. Under such conditions, safety voice requires not only hazard awareness and psychological safety, but also the ability to frame concerns appropriately, choose suitable timing, and communicate in a way that is perceived as constructive rather than confrontational. Political skill therefore functions as an interpersonal conversion capability that helps young workers transform safety concerns into acceptable and actionable upward communication (45).

#### 5.1.3 Demand-side barriers and their conditionality

A third key finding concerns the role of job insecurity and perceived voice cost. The low-safety-voice configurations show that these demand-side conditions frequently appear in silencing pathways, confirming that employment uncertainty and anticipated interpersonal or image-related risks can discourage workers from speaking up. This is consistent with earlier studies arguing that workers may remain silent when they fear retaliation, damaged relationships, or negative career consequences (41, 42).

However, the second high-safety-voice configuration shows that high safety voice may still emerge even when job insecurity and perceived voice cost are present, as long as workers simultaneously possess strong political skills, high psychological safety, and high job control. This finding supports P2 and suggests that these demand-side barriers do not inevitably lead to silence. Instead, their effects depend on whether sufficient resources (political skill, psychological safety) are available to offset them. From a JD-R perspective, this reflects a compensation logic in which selected job and personal resources buffer the negative effects of demands (23).

#### 5.1.4 Configurational pathways to low safety voice

A fourth key finding is that low safety voice is not simply the mirror image of high safety voice. The analysis identified six distinct configurations associated with low safety voice, which could be grouped into three broader patterns: a high-cost, weak-resource pathway, a resource- and capability-deficiency pathway, and a demand-loaded pathway under constrained resources (13, 55). These results support P3 by showing that low safety voice arises from specific combinations of weak resources, limited interpersonal capability, and demand-side constraints rather than from any single inhibiting condition. They also support P4 by demonstrating that the configurations associated with low safety voice differ from those associated with high safety voice rather than simply representing their opposites. This causal asymmetry reflects three distinct hindrance mechanisms: risk avoidance under high perceived voice cost, capability deficiency under weak personal and job resources, and demand accumulation when strong demands coexist with insufficient enabling resources (6, 59).

It may be explained by three underlying mechanisms. One is risk avoidance, where workers remain silent because speaking up is perceived as both unsafe and ineffective in contexts marked by low psychological safety, weak empowering supervision, and limited job control (29, 61). Another is resource and capability deficiency, in which workers may still withhold concerns even when perceived voice cost is not especially salient because they lack the political skill, supervisory support, or discretion needed to raise issues effectively (44, 59). A third is demand accumulation under constrained resources, where job insecurity and perceived voice cost jointly intensify silence when workers are already located in a weak-resource environment, consistent with the JD-R view that demands are more harmful when offsetting resources are insufficient (23, 62). Overall, these findings indicate that low safety voice should be understood as a heterogeneous configurational outcome rather than the simple opposite of high safety voice.

### 5.2 Theoretical contributions

This study makes three theoretical contributions. First, it reframes safety voice as a configurationally enabled proactive safety behavior. Prior studies have identified many antecedents of safety voice, but often treated them as independent predictors. The present findings show that safety voice among young construction workers emerges from bundles of conditions. More specifically, high safety voice depends on the joint presence of motivational permission, behavioral discretion, and interpersonal conversion capability.

Second, this study extends JD-R theory from an additive demand-resource model to a configurational explanation of socially risky proactive behavior. This differs from previous regression-, mediation-, and moderation-based studies by focusing on how demands and resources combine rather than on their independent average effects. Rather than showing that demands uniformly reduce safety voice and resources uniformly increase it, the findings reveal resource bundling, compensation, and bottleneck effects. Strong resources may jointly enable safety voice and compensate for selected demands, whereas weak resources may constrain voice even when perceived cost is not high. Political skill further functions as a personal conversion resource that helps workers translate supportive conditions into actual safety voice. These findings suggest that JD-R theory can be extended by examining how demands, job resources, and personal resources combine rather than how they operate in isolation.

Third, the study identifies political skill as a key conversion resource in young workers' safety voice. While safety voice research has often emphasized climate and leadership, the present study shows that young workers also require interpersonal capability to act within hierarchical and relationally sensitive construction settings. Political skill is therefore not merely a personal trait, but a mechanism through which safety concerns are translated into acceptable and effective upward communication.

### 5.3 Practical implications

The findings suggest that construction organizations should manage safety voice as a bundled governance issue rather than as a narrow communication problem. Encouraging young workers to speak up requires more than telling them that safety is important. Organizations need to create combinations of psychological safety, supervisory encouragement, job control, and communication capability.

For high-cost constrained voice, the priority is to reduce the anticipated interpersonal and employment consequences of speaking up. Organizations should adopt explicit non-retaliation rules and communicate them during induction, toolbox meetings, and supervisor briefings. These rules should be supported by a clear response protocol requiring supervisors to acknowledge a reported concern, assess the hazard, communicate the action taken, and explain the outcome within a specified time frame. Regular supervisor listening routines, anonymous or low-threshold reporting channels, and visible feedback boards or digital logs can further demonstrate that reported hazards lead to corrective action rather than blame. Safety reports should also be separated from decisions concerning task allocation, performance appraisal, and continued employment.

For capability- and resource-deficient low voice, the intervention should strengthen both workers' communication capability and the resources needed to act. New and young workers can be paired with experienced mentors who model how to identify, frame, and escalate safety concerns. Scenario-based communication training, role-playing, and short upward safety communication scripts can help workers state the hazard, describe the possible consequence, and request a specific corrective action. Supervisors should actively invite concerns during pre-task meetings, coach workers after voice episodes, and provide clear escalation routes and sufficient job control, including authority to pause or question work when an immediate hazard is observed.

For demand-loaded muted voice, managers should address employment insecurity and weak workplace resources simultaneously. This can be achieved by clarifying that good-faith safety reporting will not threaten employment opportunities, strengthening supervisory support, and giving workers greater discretion over safety-related actions.

Overall, these configuration-specific interventions provide a more targeted approach to improving safety voice, worker protection, and participatory safety management in construction.

### 5.4 Limitations and future research

This study has several limitations that suggest directions for future research. First, although the present study focused on young construction workers because they represent a vulnerable group in high-risk work settings, the findings were derived from a specific sample and context. Future studies could collect larger and more representative samples, including samples exceeding 500 respondents, and extend the investigation to other age groups, regions, industries, project types, employment arrangements, and cultural settings. This would help determine whether the configurational patterns identified in this study remain stable and generalizable across different contexts. Second, this study relied on cross-sectional self-reported survey data. Although this design is appropriate for identifying configurational relationships, it does not allow strong conclusions to be drawn about causal dynamics over time. Future studies could adopt longitudinal, time-lagged, or multi-source designs to further examine how safety voice develops under changing work conditions. Third, the present study examined a limited set of antecedent conditions from a JD-R perspective. Although these conditions captured several important job demands, job resources, and personal capability factors, other relevant antecedents, such as safety climate, coworker support, safety motivation, voice efficacy, and organizational trust, were not included. Future research could incorporate a broader range of conditions to provide a more comprehensive configurational explanation of safety voice.

## 6 Conclusion

This study examined safety voice among young construction workers through a configurational JD-R lens. Using fsQCA, it shows that safety voice is not driven by any single antecedent, but by multiple combinations of job demands, job resources, and personal capability. In particular, political skill emerged as a particularly important enabling condition in the configurations associated with high safety voice, while job control, psychological safety, and empowering supervision also played supportive roles across different pathways. By contrast, low safety voice was associated with several distinct combinations characterized by insufficient resources, limited capability, and, in some cases, stronger perceived constraints.

The findings further show that low safety voice is not merely the absence of high safety voice. Rather, voice and silence are generated through distinct causal pathways, highlighting the value of moving beyond linear net-effect logic when explaining proactive safety behavior in complex and high-risk construction settings. This is especially important in the construction context, where young workers' willingness to speak up may depend not only on objective workplace conditions, but also on whether they possess the interpersonal capability to express concerns effectively within hierarchical relationships.

Overall, this study contributes to the safety voice literature by offering a more realistic explanation of why young construction workers speak up or remain silent under different combinations of workplace conditions. It also extends JD-R research by showing that proactive safety behavior is shaped by configurational patterns of demands, resources, and personal capability rather than by isolated factors alone. Most importantly, the findings underscore the central role of political skill in enabling safety voice among young construction workers. From a practical perspective, fostering safety voice requires not only supportive supervision, psychological safety, and job control, but also greater managerial attention to developing young workers' ability to raise concerns appropriately and effectively in real construction site environments.

## Data Availability

The raw data supporting the conclusions of this article will be made available by the authors, without undue reservation.
